# Label-Free Ratiometric Homogeneous Electrochemical Strategy Based on Exonuclease III-Aided Signal Amplification for Facile and Rapid Detection of miR-378

**DOI:** 10.1155/2024/8368987

**Published:** 2024-05-21

**Authors:** Bingyuan Fan, Qian Wang, Shan Wang, Yahui Gao, Yan Liang, Jinru Pan, Xinrui Fu, Li Li, Wei Meng

**Affiliations:** ^1^Key Laboratory of Biomedical Functional Materials, School of Sciences, China Pharmaceutical University, Nanjing 211198, China; ^2^Nanpi No. 1 Middle School, Cangzhou 061599, China; ^3^School of Life Science and Technology, China Pharmaceutical University, Nanjing 211198, China; ^4^Nanjing Drum Tower Hospital, The Affiliated Hospital of Nanjing University Medical School, Nanjing 210008, China

## Abstract

MiR-378 is abnormally expressed in various cancers, such as hepatocellular carcinoma, renal cell carcinoma, and nonsmall cell lung cancer. Here, we developed a label- and immobilization-free ratiometric homogeneous electrochemical strategy based on exonuclease III (Exo III) for the facile and rapid determination of miR-378. Two 3′-protruding hairpin DNA probes (HPs) are designed in this strategy. Doxorubicin (DOX) and potassium ferrocyanide (Fe^2+^) were used as label-free probes to produce a response signal (I_DOX_) and a reference signal (I_Fe_^2+^) in the solution phase. When no target was present in the solution, the HP was stable, most of the DOX was intercalated in the stem of the HP, and the diffusion rate of DOX was significantly reduced, resulting in reduced electrochemical signal response. When miR-378 was present, double-cycle signal amplification triggered by Exo III cleavage was initiated, ultimately disrupting the hairpin structures of HP1 and HP2 and releasing a large amount of DOX into the solution, yielding a stronger electrochemical signal, which was low to 50 pM. This detection possesses excellent selectivity, demonstrating high application potential in biological systems, and offers simple and low-cost electrochemical detection for miR-378.

## 1. Introduction

With the continuous development of medical technology in recent years, several deadly diseases have become curable. Cancer continues to pose a serious threat to human health. In its early stages, cancer often presents with no notable symptoms. Upon diagnosis, the treatment typically includes surgery [1], radiotherapy [2], chemotherapy [3], targeted therapy [4], and immunotherapy [5]. Regardless of the treatment approach, early diagnosis is crucial for its treatment [6]. With the advancements in cancer research, new diagnostic methods are constantly being proposed, and the detection of microRNAs (miRNAs) is a highly promising avenue of research [7–9].

MiRNAs are a class of small, endogenous, noncoding RNAs that regulate target gene expression at the posttranscriptional level by binding to the 3′ untranslated region (3′ UTR). Several cancers exhibit an abnormal expression of specific miRNAs during development. Abnormal miR-378 expression has been reported to be associated with several types of cancers, including hepatocellular carcinoma [10], renal cell carcinoma [11], and nonsmall-cell lung cancer [12]. However, owing to the low expression levels of miRNAs, using highly sensitive detection methods is necessary.

Electrochemical detection, which is characterized by high sensitivity, rapid response, good selectivity [13], and simplicity of operation, has been employed by researchers for microRNA detection [14–20]. However, conventional heterogeneous electrochemical biosensors typically involve the immobilization of a probe on a working electrode, which requires laborious and time-consuming electrode pretreatment and probe immobilization steps [21–25]. Moreover, the efficiency of probe immobilization may vary after electrode pretreatment, hindering the reproducibility of the test results. In addition, probes situated in the same plane may face limitations due to steric hindrance, which subsequently decreases the effectiveness of recognition and binding. By contrast, homogeneous electrochemical biosensors, whose recognition and response processes occur in the solution phase instead of on the electrode surface, avoid complicated immobilization processing [26–29].

Currently, most homogenous electrochemical sensors use DNA probes labeled with methylene blue (MB) as signal-responsive substance [30–32]. However, these labeled probes are time consuming and costly. Moreover, methylene blue, a dye molecule, exhibits significant adsorption properties that could affect the accuracy of the test results. DOX can specifically intercalate into dsDNA without strong nonspecific adsorption [33].

Ratio electrochemical sensors utilize two independent response signals to compare and detect targets, effectively eliminating the interference caused by external factors and improving the accuracy and reliability of the results [34, 35]. Chen et al. developed an electrochemical aptamer-based biosensor for a highly sensitive thrombin assay using MB and ferrocene (Fc) as electrochemically active markers, which exhibited different signal changes in the presence of thrombin. Through amplification of the ratio signal, high-sensitivity detection of thrombin was achieved with a detection limit as low as 56 fM [36]. Zhu et al. utilized aptamer and hybrid chain reactions using Fc as an internal standard to achieve the highly sensitive detection of aflatoxin B1 based on the ratio of methylene blue to Fc signals [37].

In this study, we developed a label-free homogeneous electrochemical sensor for miR-378 determination based on exonuclease III (Exo III)-assisted recycling amplification. HP1 and HP2 were designed as 3′-protruding structures. In the absence of the target, DOX in the solution was embedded in the stem-loop structures of HP1 and HP2, thus reducing its diffusion rate in the solution and exhibiting a lower electrochemical signal. When miR-378 is present, it triggers the cleavage process of Exo III. Ultimately, the stem-loop structures of HP1 and HP2 were disrupted, releasing a large amount of DOX into the solution, resulting in a higher electrochemical signal. Throughout this process, the electrochemical signal of Fe^2+^ remained relatively constant. miR-378 was achieved by calculating the ratio of DOX to Fe^2+^ current. This method allows for the sensitive, rapid, and accurate determination of miR-378.

## 2. Experiment

### 2.1. Reagents

Magnesium chloride, potassium chloride, sodium hydroxide, and sodium chloride were purchased from Sinopharm Chemical Reagents (China). DOX and potassium ferrocyanide were purchased from Aladdin Chemical (Shanghai, China). Agarose, 50× TAE buffer, Tris(hydroxymethyl)aminomethane (Tris), Exo III, Fetal bovine serum, and all oligonucleotides are from Sangon Biotech (China). The sequence is listed in Table S1.

Exo III reaction buffer (10 mM Tris HCl, 10 mM MgCl_2_, 10 mM KCl, 50 mM NaCl, and pH 7.6). Milli-Q water was used for all of the solutions.

### 2.2. Instrumentation

All of the electrochemical measurements were carried out on a CHI 660C workstation (Chenhua, Shanghai) with a three electrode system of an indium tin oxide (ITO) working electrode, a platinum wire counter electrode and a reference electrode of Ag/AgCl (sat. KCl). Differential pulse voltammetry (DPV) was performed at a scanning potential from −0.7 to 0.6 V with an increment potential of 4 mV, the amplitude, the pulse width, and the pulse period were set to values of 0.05 V, 0.05 s, and 0.5 s. Before measurement, the ITO electrode was treated with water ethanol and acetone for 10 min repeated. Then, the ITO electrode was immersed in 1 mM NaOH solution for 5 h and sonicated in ultrapure water for 10 min. Finally, a negatively charged working electrode surface was obtained [38].

### 2.3. Construction and Detection Process of This Assay

HP was preheated to 95°C for 5 min and cooled to room temperature before use. Then, a mixture of 100 *μ*L containing 0.7 *μ*M HP1, 3.5 *μ*M HP2, 0.16 unit/*μ*L Exo III, and varying concentrations of target DNA in a 10 mM Tris-HCl buffer was incubated at 37°C for 75 min; subsequently, add 100 *μ*L of the sample to 1 mL of 10 mM Tris HCl buffer containing 0.4 *μ*M DOX and 10 *μ*M Fe^2+^. The solution was mixed at room temperature in the dark for 20 min before electrochemical testing.

### 2.4. Gel Electrophoresis

Gel electrophoresis was conducted on a 5% agarose gel at room temperature in TAE buffer at 120 mA for 60 min. The red-stained gel was imaged using a Tanno 1600+ Imaging System (Tanon, China).

### 2.5. Cell Culturing and Extraction of miRNA-378

Human normal liver cells (L02) and human cervical cancer cell lines (HeLa) were cultured in Dulbecco's modified Eagle's medium supplemented with 10% fetal bovine serum at 37°C in a 5% CO_2_ atmosphere. During the exponential phase of growth, cells were collected and washed twice with phosphate buffered saline (pH 7.4), thereafter suspended in 1× CHAPS lysis buffer, shaken at 4°C for 30 min, and centrifuged at 10,000 rpm for 30 min at 4°C. The resulting supernatant was collected and stored at −80°C until used for further analysis.

## 3. Results and Discussion

### 3.1. Design Principle of the Sensor

Here, we report a label-free homogeneous electrochemical sensing platform for an ultrasensitive miR-378 assay based on Exo III-aided recycling amplification.

**Scheme 1:**
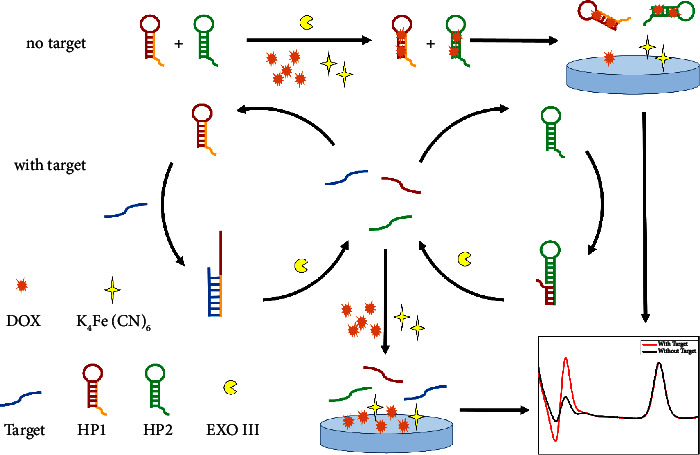
Schematic of exo III-assisted label-free homogeneous electrochemical strategy for ultrasensitive detection of miR-378.

The nucleic acids in this detection system typically contained hairpin probes HP1 and HP2. The HP could form a stable stem-loop structure, including a 3′-protruding DNA fragment. As shown in Scheme 1, in the absence of miR-378, the HP was stable, most of the DOX was intercalated into the stem of the HP, and the diffusion rate of DOX was significantly reduced. Furthermore, owing to the electrostatic repulsion of the HP towards the indium tin oxide (ITO) electrode, the intercalated DOX molecules could not easily reach the electrode surface, resulting in a reduced electrochemical signal response.

When miR-378 is present, HP1 binds to miR-378, forming a 3′ terminus recognized by the Exo III. Exo III is then triggered to cleave HP1, releasing both miR-378 and the DNA fragment of HP1. The released miR-378 then binds to the next HP1, whereas the remaining DNA fragment of HP1 binds to HP2, triggering endonuclease III. Thus, a single miRNA, miR-378, can repeatedly and consecutively trigger a signaling response.

Ultimately, the stem-loop structures of HP1 and HP2 are disrupted, releasing a large amount of DOX into the solution, resulting in a higher electrochemical signal. Fe^2+^ maintained its free state in solution throughout the reaction, and a constant I_Fe_^2+^ was used as an internal reference signal to enhance the reliability of this sensing system.

Consequently, the Exo III-based label-free ratiometric homogeneous electrochemical biosensor for detecting the miR-378 was developed because of two signal variations.

### 3.2. Feasibility of Homogeneous Electrochemical-Based miR-378 Assay

**Figure 1:**
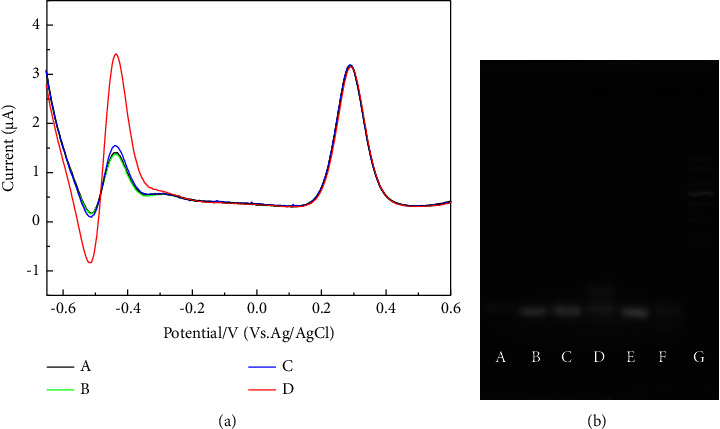
DPV curves of DOX and potassium ferricyanide under different conditions: (A) DOX + Fe^2+^ + HP, (B) DOX + Fe^2+^ + HP + miR-378, (C) DOX + Fe^2+^ + HP + exo III, and (D) DOX + Fe^2+^ + HP + exo III + miR-378 (a). Gel electrophoresis image of (lane A) HP1, (lane B) HP2, (lane C) HP1 + HP2, (lane D) HP1 + HP2 + miR-378, (lane E) HP1 + HP2 + exo III, (lane F) HP1 + HP2 + miR-378 + exo III, and (lane G) marker (b).

As shown in Figure 1(a), the signal changes in DOX under different conditions were analyzed. Upon adding HP to the system, DOX was intercalated into the HP, exhibiting a lower peak current. Furthermore, upon the addition of both HP and the target, the peak current remained largely unchanged compared to that when only HP was present. In the absence of the target, the addition of HP and Exo III resulted in a slight increase in the DOX peak current, possibly due to nonspecific cleavage by Exo III. Only when HP, the target, and Exo III were simultaneously present in the system, a significant increase in the peak current occurred.

Gel electrophoresis experiments were also performed to verify Exo III-aided target recognition in this homogeneous system. As shown in Figure 1(b), the migration of HP1 cells (lane a) was slower than that of HP2 cells (lane b). No new bands appeared in the presence of HP1 + HP2 (lane c), indicating the stable coexistence of HP1 and HP2 in the absence of the target. With the addition of miR-378 to HP1 + HP2 (lane d), a band with a molecular weight greater than that of HP1 emerged, indicating successful binding between HP1 and miR-378. In the presence of HP1 + HP2 + Exo III (lane e), no significant change in band intensity was observed, suggesting that Exo III was not triggered without a target. However, the band nearly disappeared when miR-378 and Exo III were added to the HP1 + HP2 (lane f), indicating the successful initiation of the cleavage process for Exo III.

### 3.3. Optimization of Experimental Conditions

**Figure 2:**
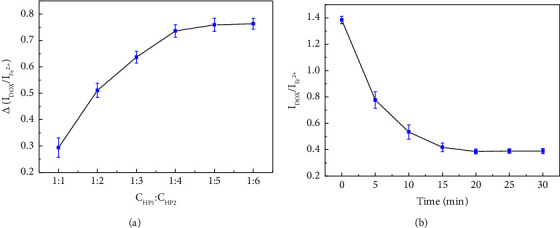
Change of I_DOX_/I_Fe_^2+^ under different ratios of HP1 and HP2 (a). The DOX signal fluctuates with the embedding time (b).

This experiment involved two hairpin probes, with HP1 primarily responsible for target recognition and HP2 amplifying the signal. The concentration ratio of the two probes influences the sensitivity of the experiment [39, 40]. Under the conditions of HP1-HP2 total concentration 2.1 *μ*M, target concentration 5 nM, DOX 1.0 *μ*M, and Fe^2+^ 10 *μ*M, electrochemical tests were conducted at 37°C for 30 min with the addition and without the addition of 0.2 U/*μ*L Exo III. Subsequently, the Δ (I_DOX_/I_Fe_^2+^) for both cases was calculated. As shown in Figure 2(a), results demonstrated that no noticeable change was observed beyond a 1 : 5 ratio of HP1-HP2. Therefore, a concentration ratio of 1 : 5 was used in the subsequent experiments.

Optimization was performed under specific experimental conditions to achieve the optimal performance. As shown in Figure S1, the initial signal of DOX at different concentrations is denoted as *I*_0_, whereas the signal after the addition of HP at different concentrations is denoted as *I*_1_. Conditions with the greatest variation relative to the blank were obtained. The ratio of *I*_0_–*I*_1_ was investigated when different concentrations of DOX were added to varying concentrations of the probes.

At a constant HP concentration, as the concentration of DOX increased, the magnitude of the signal initially increased and then decreased. This phenomenon can be attributed to excessive DOX reducing the proportion of DOX signals embedded in the double-stranded structure, whereas insufficient DOX fails to respond promptly after HP cleavage, thereby lowering the sensitivity of the detection system. At a constant DOX concentration, as the concentration of hairpin probes increased, the magnitude of the signal change increased. However, excess hairpin probes can affect the cleavage time of Exo III. Considering the probe dosage and subsequent enzyme cleavage reaction, the experimental conditions were ultimately selected as DOX 0.4 *μ*M, HP1 0.7 *μ*M, and HP2 3.5 *μ*M. The effect of HP on the Fe^2+^ signals was tested under these conditions, as shown in Figure S3. No significant differences were observed in the signals before and after the addition of HP.

After determining the probe concentration (Figure 2(b)), the intercalation times for HP and DOX were optimized. After 20 min, no significant signal changes were observed, indicating complete binding between DOX and HP.

**Figure 3:**
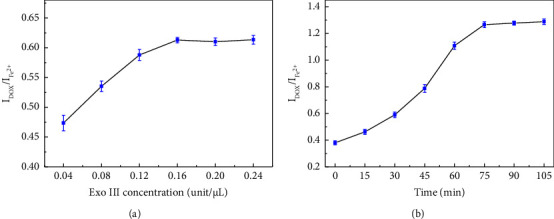
Optimization of exo III concentration (a) and the reaction time (b).

After determining the concentration of the HP, the enzyme digestion conditions for Exo III were optimized. Under the aforementioned experimental conditions, 5 nM miR-378 and varying concentrations of Exo III were added, followed by incubation at 37°C for 30 min.  Figure 3(a) shows that the electrochemical signal no longer increased significantly at 0.16 U/*μ*L [41–43]. The digestion time was further optimized. As shown in Figure 3(b), the electrochemical signal ceased to increase after 75 min. In addition, further optimizations were made for digestion temperature (Figure S4) and pH (Figure S5).

### 3.4. Analytical Performance of the Assay

**Figure 4:**
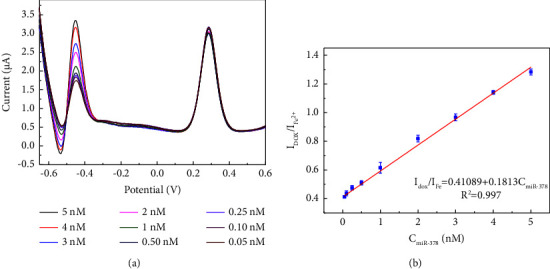
DPV responses towards miR-378 with different concentrations (5, 4, 3, 2, 1, 0.5, 0.25, 0.1, and 0.05 nM) (a). The calibration curve for miR-378 determination (plot of I_DOX_/I_Fe_^2+^ vs. concentrations of the miR-378) (b).

Under optimized experimental conditions, the DPV responses were tested with a series of concentrations of miR-378. As shown in Figure 4(a), the DPV signals increased successively with increasing miR-378 concentration, indicating a strong dependence of the electrochemical signal of DOX on the concentration of the target DNA. This confirms the working principle of triggering Exo III digestion by hybridizing the target DNA with the hairpin probe.  Figure 4(b) demonstrates an excellent linear correlation (correlation coefficient of 0.997) between I_DOX_/I_Fe_^2+^ and target concentrations ranging from 0.05 to 5 nM. Direct measurement of the target DNA achieved a detection limit as low as 50 pM.

### 3.5. Selectivity, Reproducibility, and Stability of the Assay

**Figure 5:**
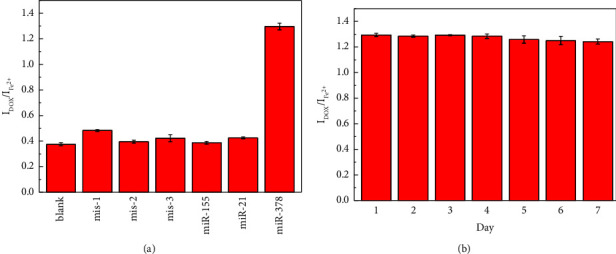
Selectivity of the developed biosensor to miR-378, mis-1, mis-2, mis-3, miR-155, miR-21, and blank (a). The stability of the proposed assay for 7 days (b).

To assess the selectivity of the proposed method, interference tests using other miRNAs and mismatched sequences with miR-378 were performed at a concentration of 5 nM for both the interfering agents and the target [44].  Figure 5(a) illustrates that the signals from the interfering agents showed little to no discernible difference from the blank, whereas the presence of miR-378 elicited a notable response, indicating the excellent selectivity of the assay. Furthermore, as shown in Figure S2, the reproducibility of the measurements was evaluated by performing parallel measurements of miR-378 at 5 nM nine consecutive times [45], which yielded a relative standard deviation estimate of 1.18%. In addition, to assess stability, the samples were stored in the dark at 4°C for 7 days and tested daily. As shown in Figure 5(b), even after 7 days, the DPV response retained 96.0% of the initial signal after storage at 4°C for 7 days indicating good stability [46].

### 3.6. miRNA Detection in the Sample

To evaluate the applicability of the assay, varied concentrations of miRNA-378 were introduced into bovine serum diluted at a ratio of 1 : 20. Preprocessing involves thawing the frozen plasma to room temperature and diluting it with a buffer before spiking [47]. As shown in Table 1, the recovery rates ranged from 99.65% to 102.56%. These results demonstrate promising prospects for detecting miRNAs in authentic biological samples.

Moreover, the expression level of miRNA-378 in cancer cell was further studied with this electrochemical biosensor. The cell lysates were extracted from HeLa cells and L02 cells and used after 50-fold dilution. The content of miR-378 is higher in HeLa cells than in normal cells (Figure S6). This results consistent with the earlier reported [48] and indicate the potential application in complex sample detection.

## 4. Conclusion

In summary, a label-free and immobilization-free ratiometric homogeneous electrochemical biosensor based on Exo III was developed. The Exo III-aided method achieved target recycling signal amplification, enabling the rapid and simple detection of miR-378. Compared to other miRNA detection methods, this method avoids probe modification. DOX and Fe^2+^ can simultaneously generate response signal (I_DOX_) and reference signal (I_Fe_^2+^) in solution. The miR-378 can be quantified by the ratio of I_DOX_/I_Fe_^2+^, reducing the influence of the testing environment on the results and improving the accuracy of the test results. The detection process of this method was conducted in solution, avoiding the intricate modification and probe immobilization steps of heterogeneous electrochemical sensors. Therefore, this strategy holds great promise as a simple and highly sensitive method for miR-378 detection and demonstrates significant practical application prospects for early cancer diagnosis based on miR-378.

## Figures and Tables

**Table 1:** Determination of miRNA-378 in bovine serum sample with suggested methodology.

Sample	Added (nM)	Founded (nM)	Recovery (%)
1	2.5	2.49	99.65
2	10	10.26	102.56
3	25	25.46	101.84

## Data Availability

The data used to support the findings of this study are available from the corresponding author upon reasonable request.

## Supplementary Materials

Supplementary Materials Table S1: oligonucleotides used in this research. Figure S1: optimization of HPDNA and DOX concentration conditions. Figure S2: the reproducibility by 7 parallel measurements of 50 nM miR-378. Figure S3: the signal of HPDNA affects the Fe^2+^. Figure S4: the I_dox_/I_Fe_ at different temperatures. Figure S5: the I_dox_/I_Fe_ at different pH values. Figure S6: expression of miRNA in different cell extracts. Figure S7: the MB signal at different adsorption time.
