# Supplementary material for: Label-Free Ratiometric Homogeneous Electrochemical Strategy Based on Exonuclease III-Aided Signal Amplification for Facile and Rapid Detection of miR-378
Source: Int J Anal Chem. 2024 May 21;2024:8368987. doi: 10.1155/2024/8368987 (PMC11132827; doi:10.1155/2024/8368987)
Supplement: Supplementary Materials — Table S1: oligonucleotides used in this research. Figure S1: optimization of HPDNA and DOX concentration conditions. Figure S2: the reproducibility by 7 parallel measurements of 50 nM miR-378. Figure S3: the signal of HPDNA affects the Fe2+. Figure S4: the Idox/IFe at different temperatures. Figure S5: the Idox/IFe at different pH values. Figure S6: expression of miRNA in different cell extracts. Figure S7: the MB signal at different adsorption time. [file 8368987.f1.docx]

Supporting information

**Label-free ratiometric homogeneous electrochemical strategy based on Exonuclease Ⅲ-Aided signal amplification for facile and rapid detection of miR-378**

Bingyuan Fan ^a^, Qian Wang ^a^, Shan Wang ^a^, Yahui Gao ^a^, Yan Liang ^a^, Jinru Pan ^a^, Xinrui Fu ^b^, Li Li ^c,^ *, Wei Meng ^a,^ *

^a^ Key Laboratory of Biomedical Functional Materials, School of Sciences, China Pharmaceutical University, Nanjing, Jiangsu, China;

^b^ School of Life Science and Technology, China Pharmaceutical University, Nanjing 211198, China;

^c^ Nanjing Drum Tower Hospital, The Affiliated Hospital of Nanjing University Medical School, Nanjing 210008, China;

^∗^ Corresponding authors.

E-mail address: njgllily@163.com (L.li), mengwei@cpu.edu.cn (W. Meng)

Table S1. Oligonucleotides used in this research

| **Oligonucleotides** | **Sequence (5’-3’)** |
| --- | --- |
| HP1 | **GACTTGGAG**TCAGAAGAAGAA*CTGACTCCAAGTCCAGT* |
| HP2 | GCACGACGCATCATCACGATGCGTCGTGC**CTCCAAGTC** |
| hsa-miR378a-3p | *ACUGGACUUGGAGUCAG*AAGGC |
| miR-378-mis1 | ACUGGACUUG**A**AGUCAGAAGGC |
| miR-378-mis2 | ACUGGAC**A**UGGAGUCA**C**AAGGC |
| miR-378-mis3 | ACUGGAC**A**UGG**U**GUCA**C**AAGGC |
| miR-21 | UAGCUUAUCAGACUGAUGUUGA |
| miR-155 | UUAAUGCUAAUCGUGAUAGGGGU |


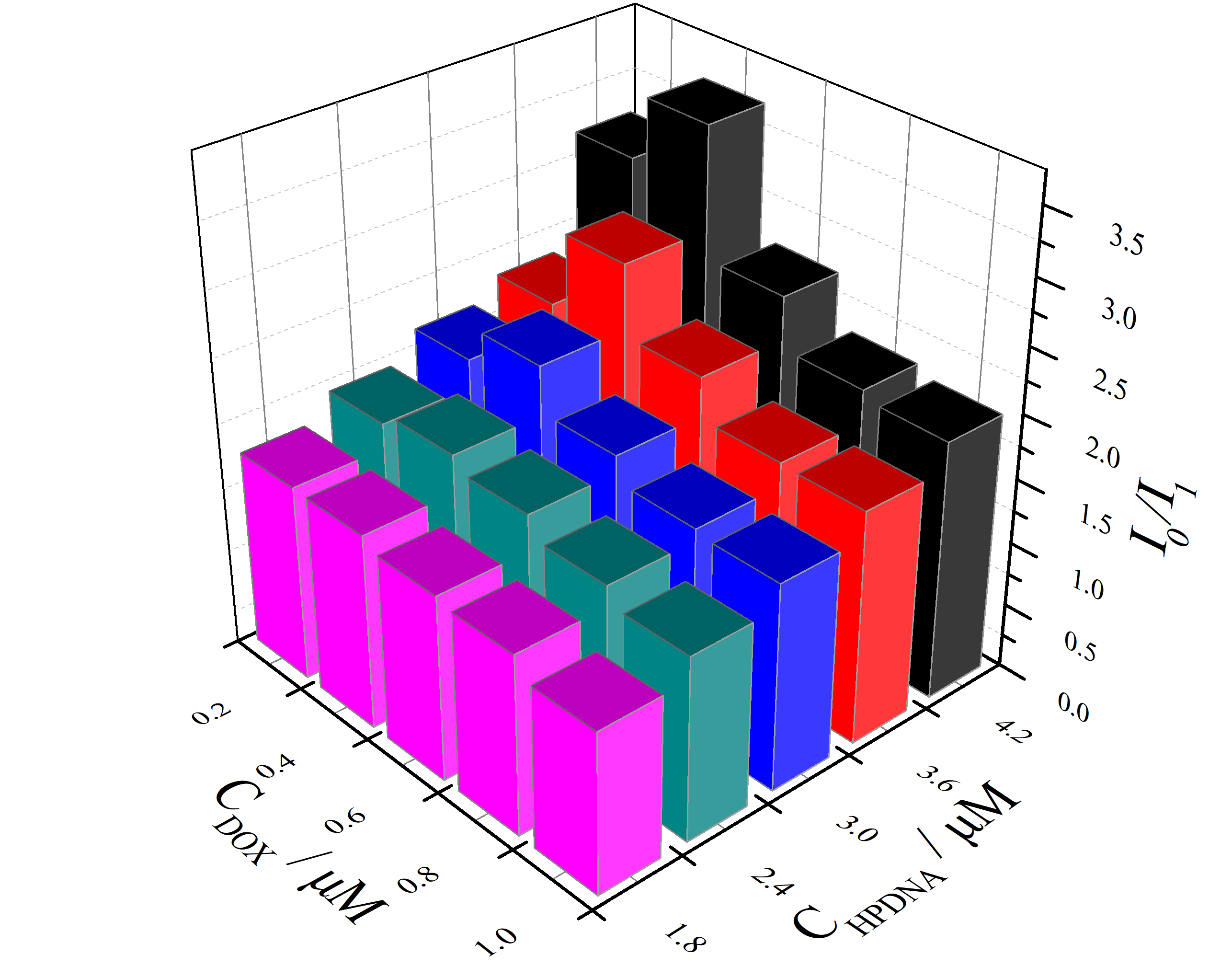


Fig. S1. Optimization of HPDNA and DOX concentration conditions.


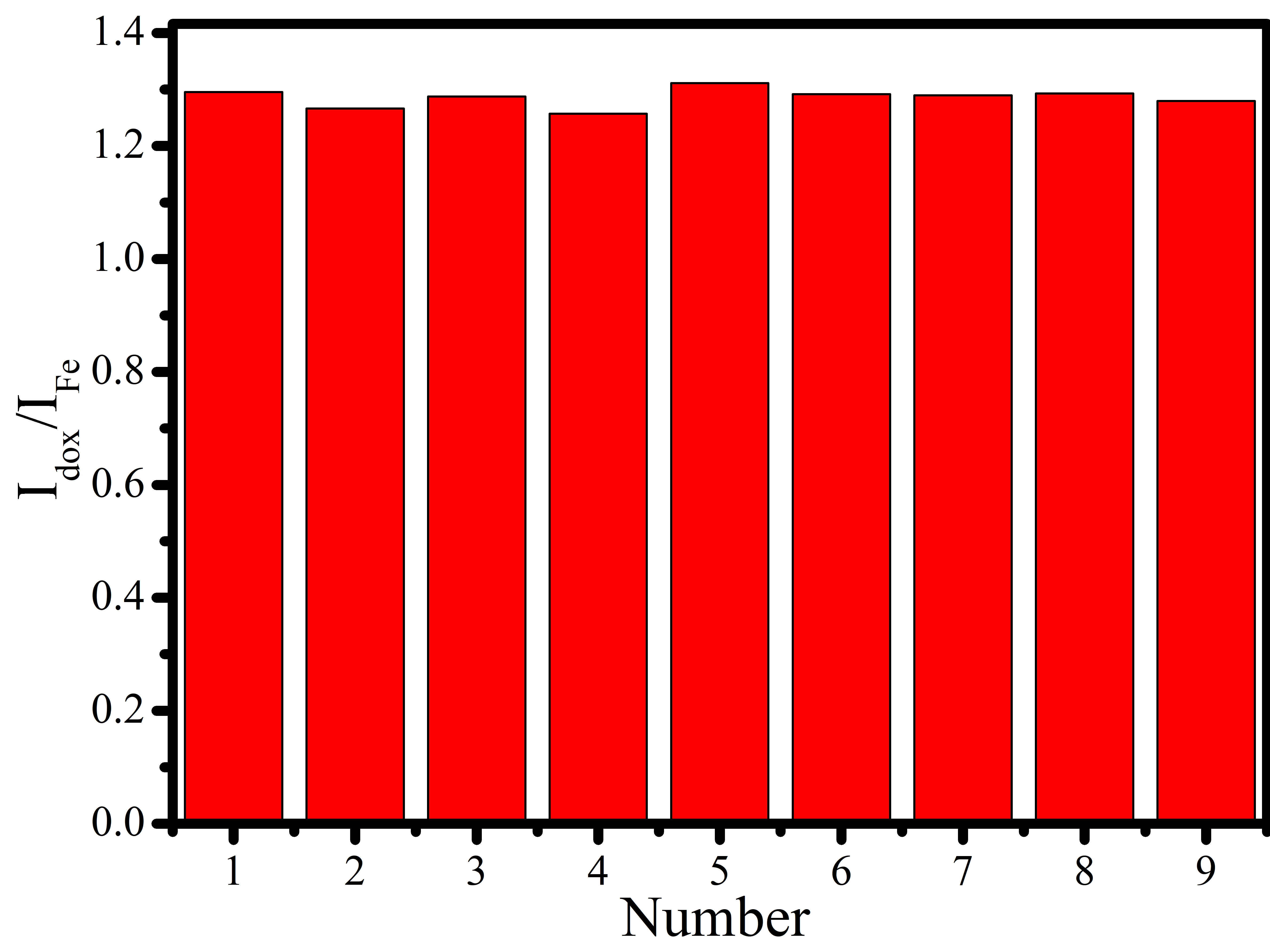


Fig. S2. The reproducibility by 7 parallel measurements of 50nM miR-378.


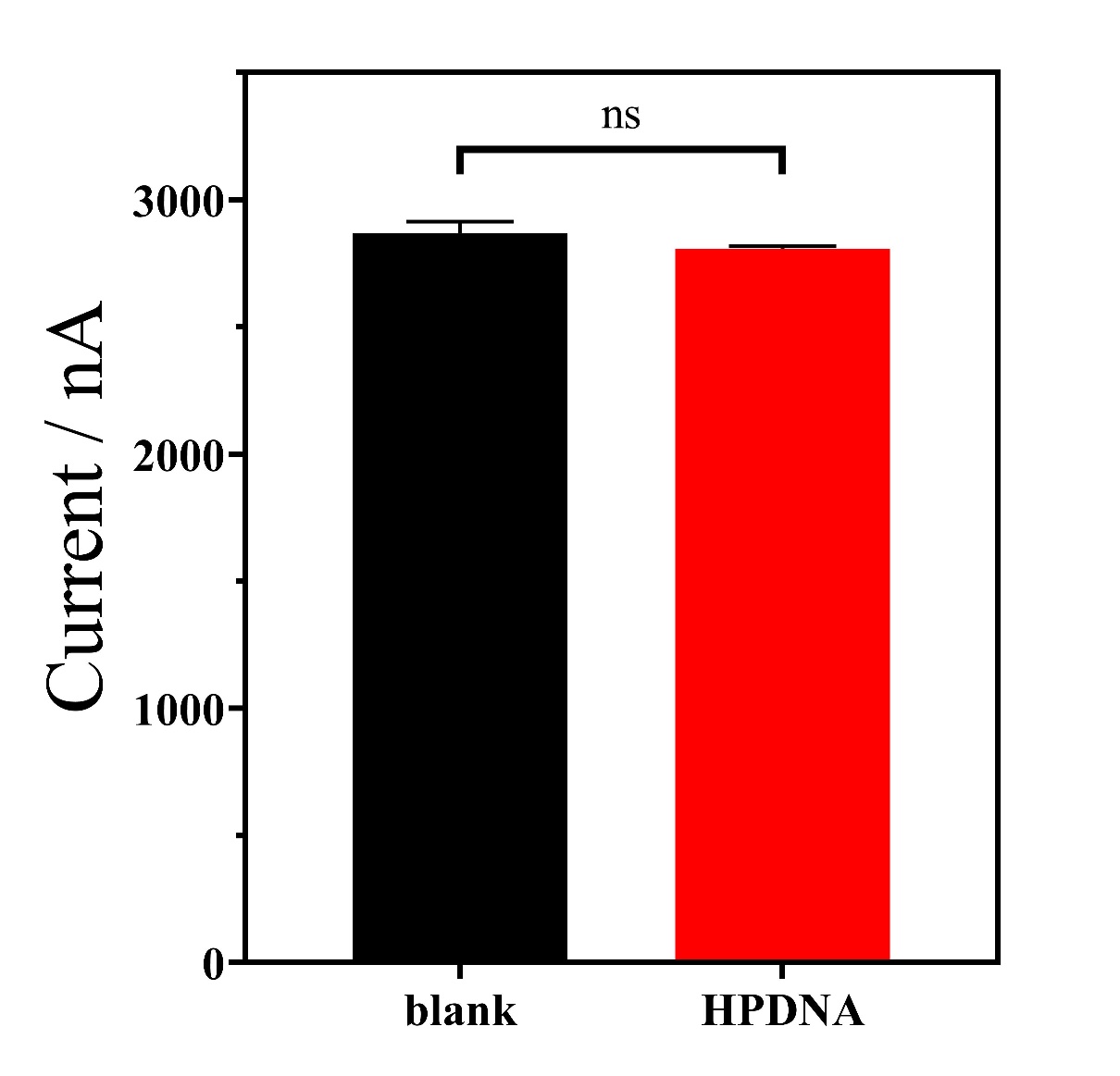


Fig. S3. The signal of HPDNA affects the Fe^2+^.


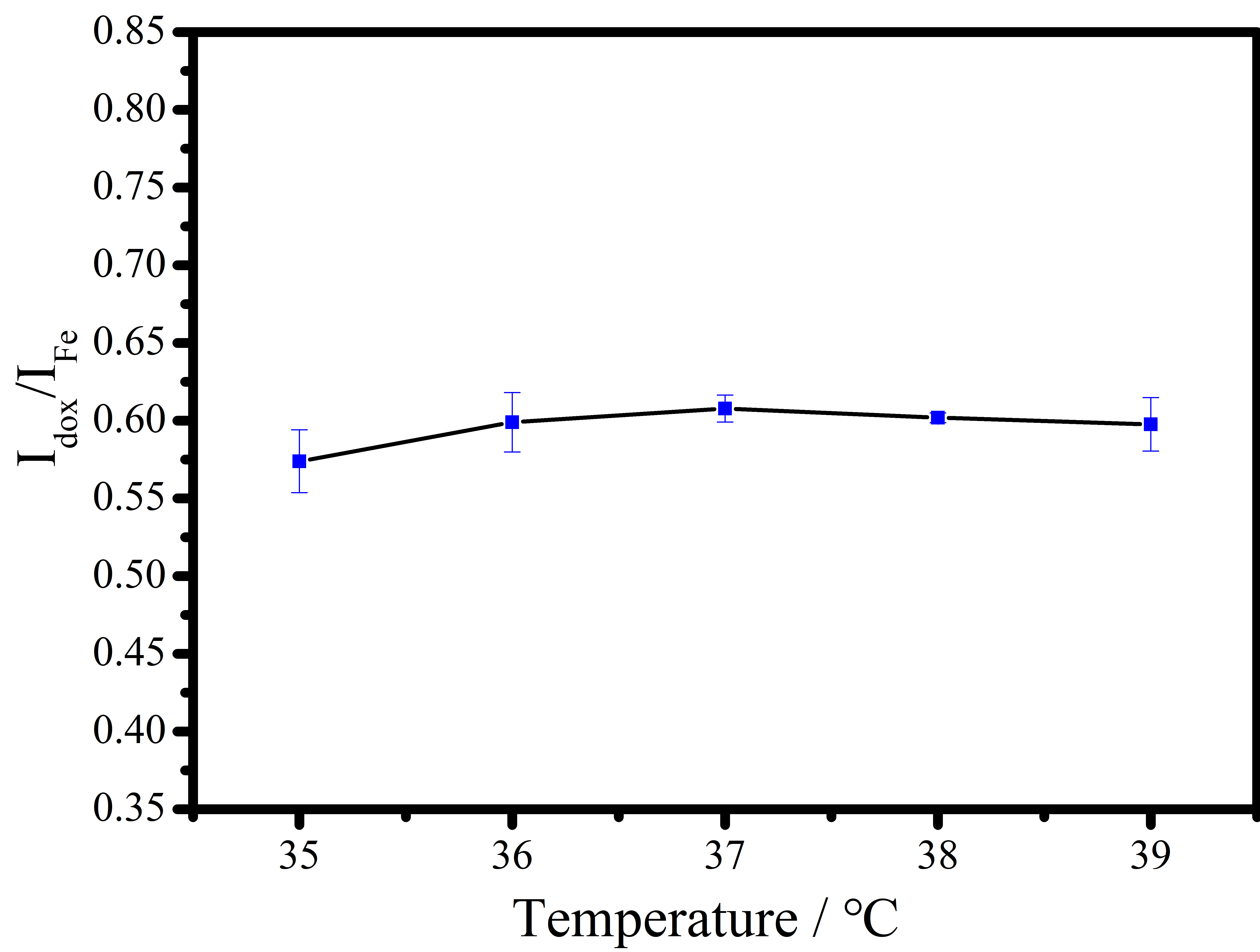


Fig. S4. The I_dox_/I_Fe_ at different temperatures.


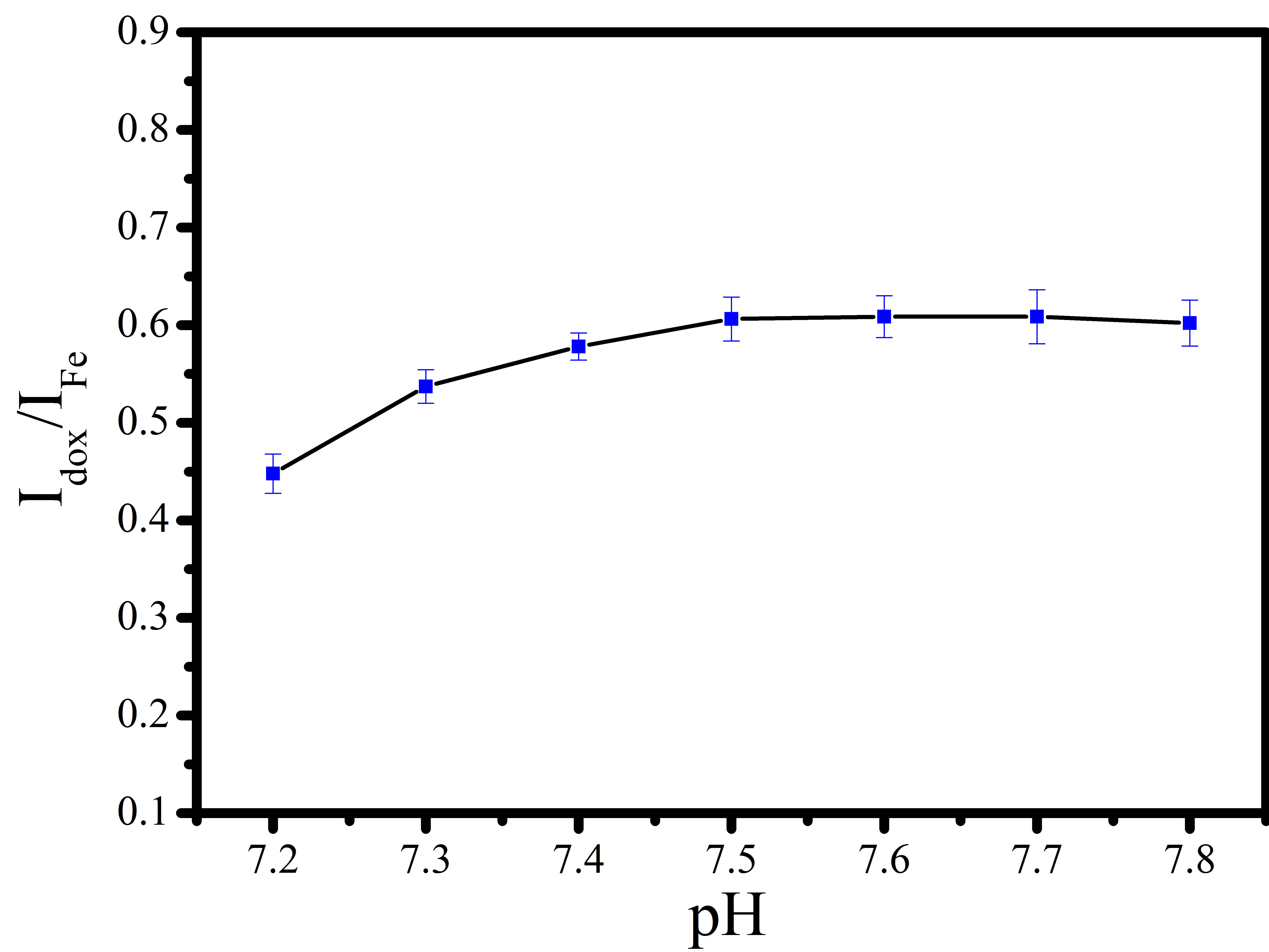


Fig. S5. The I_dox_/I_Fe_ at different pH values.

Fig.S6. Expression of miRNA in different cell extracts.


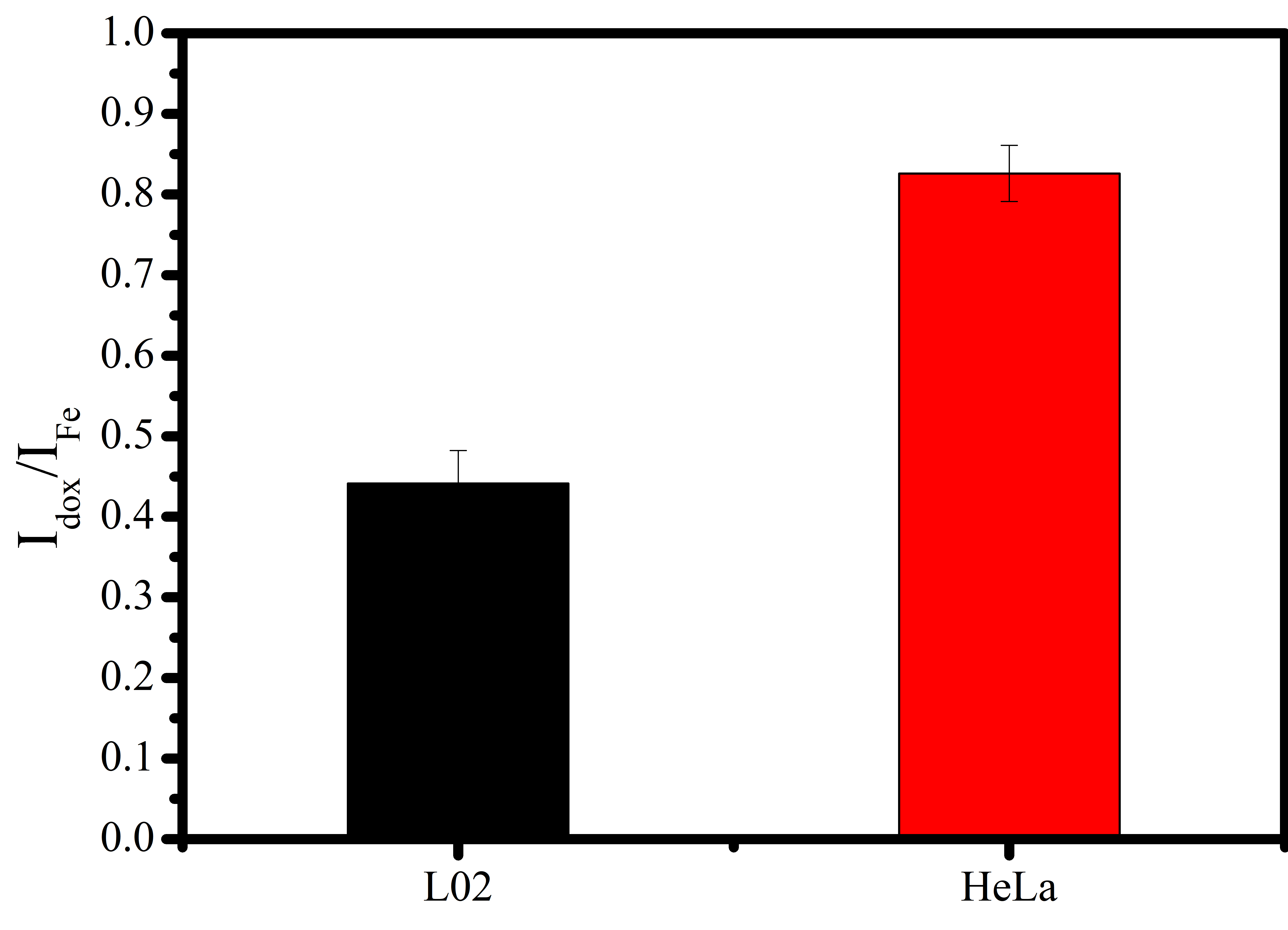

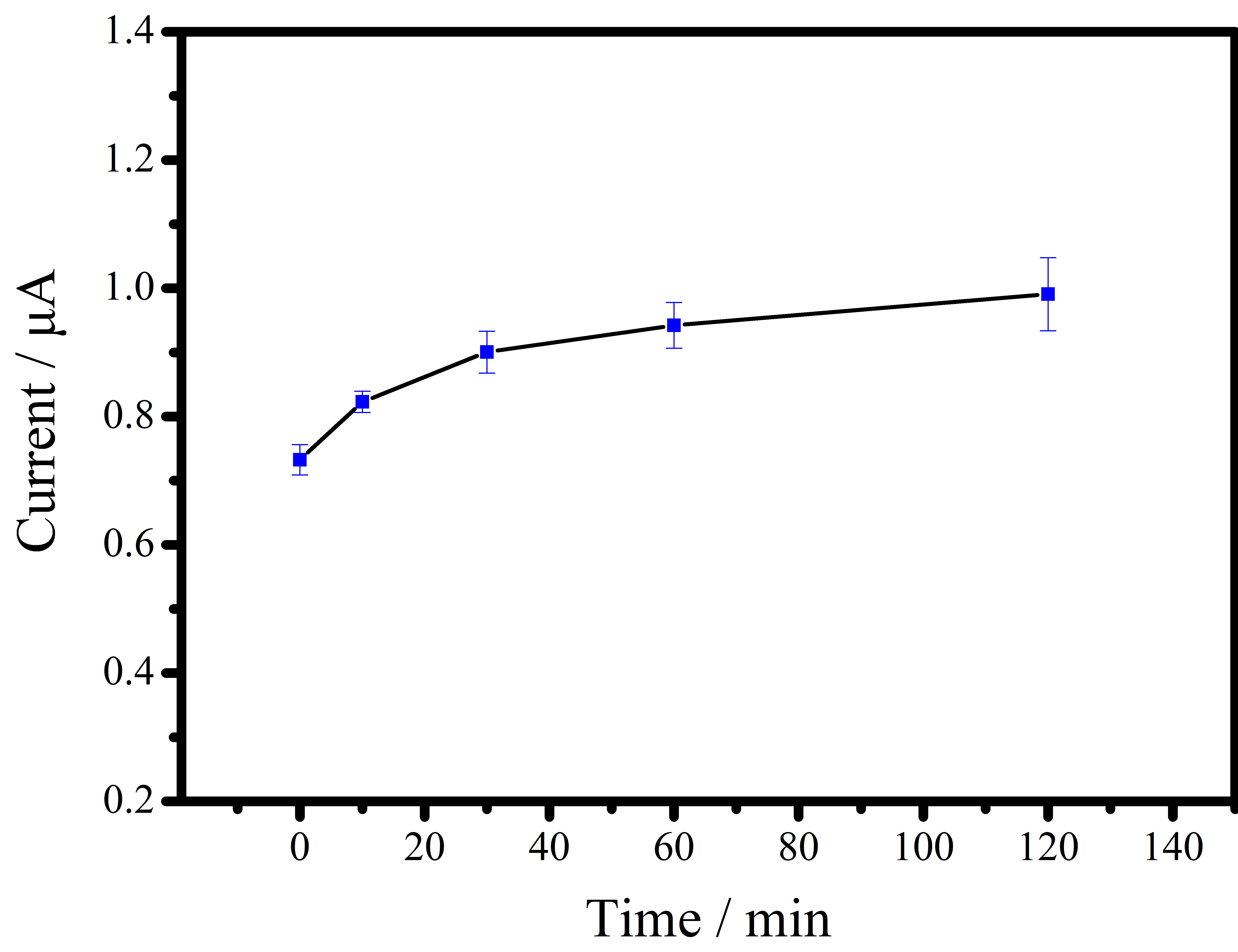


Fig. S7. The MB signal at different adsorption time.

Compared to DOX, MB has a stronger adsorption capacity. Figure S7 shows the relationship between the electrochemical signal of MB and the adsorption time on the ITO electrode in a 1μM MB solution after different immersion times. With increasing adsorption time, the electrochemical signal of MB shows a significant increase.
